# Supplementary material for: Molecular Characteristics, Receptor Specificity, and Pathogenicity of Avian Influenza Viruses Isolated from Wild Ducks in Russia
Source: Int J Mol Sci. 2022 Sep 16;23(18):10829. doi: 10.3390/ijms231810829 (PMC9502348; doi:10.3390/ijms231810829)
Supplement: Supplementary file 1 [file ijms-23-10829-s001.zip › Tables S1 and S2.pdf]

Table S1. List of viruses. Fully sequenced isolates.

Isolation data	virus	designation	subtype	Sequence Accession
04.10.2006	A/gull/Moscow/3100/06	g/3100/06	H6N2	EU152234- EU152241
13.10.2008	A/duck/Moscow/3554/2008	d/3554/08	H3N1	GU991376
13.10.2008	A/duck/Moscow/3556/2008	d/3556/08	H3N1	MN692225- MN692232
21.10.2008	A/duck/Moscow/3661/2008	d/3661/08	H4N6	MF680290- MF680297
04.11.2008	A/duck/Moscow/3641/2008	d/3641/08	H11N9	MT773558- MT773564<br>GU991377
26.08.2009	A/duck/Moscow/3740/2009	d/3740/09	H4N6	MF422098- MF422104
04.09.2009	A/duck/Moscow/3799/2009	d/3799/09	H4N6	MF422105- MF422111
30.09.2009	A/duck/Moscow/3735/2009	d/3735/09	H4N6	MF422091- MF422097
04.09.2009	A/duck/Moscow/3806/2009	d/3806/09	H3N8	MN692266-MN692273
21.09.2009	A/duck/Moscow/3720/2009	d/3720/09	H6N2	MW269601- MW269607<br>CY120771
26.11.2010	A/duck/Moscow/4203/2010	d/4203/10	H3N8	MN700132- MN700139
16.10.2010	A/duck/Moscow/4238/2010	d/4238/10	H3N6	MN700140- MN700147
13.11.2010	A/duck/Moscow/4242/2010	d/4242/10	H3N8	MN700148- MN700155
22.10.2010	A/duck/Moscow/4298/2010	d/4298/10	H3N8	MN700156- MN700163
20.09.2010	A/duck/Moscow/4031/2010	d/4031/10	H6N2	MT773263- MT773270
16.11.2010	A/duck/Moscow/4182/2010	d/4182/10	H5N3	KF885672- KF885679
27.09.2011	A/duck/Moscow/4494/2011	d/4494/11	H3N8	MN759696- MN759703
21.10.2011	A/duck/Moscow/4681/2011	d/4681/11	H3N8	MN759704- MN759711
04.10.2011	A/duck/Moscow/4518/2011	d/4518/11	H4N6	MF673524- MF673531
04.10.2011	A/duck/Moscow/4524/2011 mix	d/4524/11<br>mix	H3N2<br>H3N8	MN692213- MN692221<br>MN692219(N8)
04.10.2011	A/duck/Moscow/4528/2011	d/4528/11	H4N6	MF673532- MF673539
19.10.2011	A/duck/Moscow/4641/2011	d/4641/11	H4N6	MF422112- MF422119
19.10.2011	A/duck/Moscow/4643/2011	d/4643/11	H4N6	KX509943- KX509950
11.10.2011	A/duck/Moscow/4652/2011	d/4652/11	H4N6	KX518711- KX518718
11/.10.2011	A/duck/Moscow/4661/2011	d/4661/11	H3N8	MN759712- MN759719
10.10.2012	A/duck/Moscow/4771/2012	d/4771/12	H4N6	MF673540- MF673547
17.10.2012	A/duck/Moscow/4843/2012	d/4843/12	H4N6	MF673548- MF673555
17.10.2012	A/duck/Moscow/4788/2012	d/4788/12	H3N8	MT773383- MT773390
31.10.2012	A/duck/Moscow/4780/2012	d/4780/12	H3N8	MT773360- MT773367
31.10.2012	A/duck/Moscow/4781/2012	d/4781/12	H4N6	KX530510- KX530517
19.11.2013	A/duck/Moscow/4971/2013	d/4971/13	H5N3	MN588283- MN588290
26.11.2013	A/duck/Moscow/4952/2013	d/4952/13	H5N3	MN588194- MN588201
26.11.2013	A/duck/Moscow/4970/2013	d/4970/13	H1N1	MN400364- MN400371
26.10.2014	A/duck/Moscow/5037/2014	d/5037/14	H3N8	MT773420- MT773427
04.09.2015	A/duck/Moscow/5163/2015	d/5163/15	H3N6	OP132933-OP132940
16.09.2015	A/duck/Moscow/5169/2015	d/5169/15	H3N6	OP133010-OP133017
07.09.2015	A/duck/Moscow/5171/2015	d/5171/15	H3N6	OP133377-OP133384
07.09.2015	A/duck/Moscow/5172/2015	d/5172/15	H3N6	OP133387-OP133394
17.10.2018	A/duck/Moscow/5586/2018	d/5586/18	H1N2	MN435632- MN435639
01.11.2018	A/duck/Moscow/5662/2018	d/5662/18	H1N2	MN588291- MN588298
15.10.2019	A/duck/Moscow/5743/2019	d/5743/19	H1N1	MW186793- MW186800
15.10.2019	A/duck/Moscow/5744/2019	d/5744/19	H1N1	MW186801- MW186808
21.10.2019	A/duck/Moscow/5712/2019	d/5712/19	H11N6	MW186784- MW186791
06.10.2021	A/duck/Moscow/5881/2021	d/5881/21	H3N8	OP133621-OP133628
07.11.2021	A/duck/Moscow/5897/2021	d/5897/21	H3N8	OP136008-OP136015
07.11.2021	A/duck/Moscow/5908/2021	d/5908/21	H3N8	OP135948-OP135955

Table S2. List of viruses. Partly sequenced isolates.

**26.11.2010**	**A/duck/Moscow/4206/2010**	**d/4206/10**	**H5N3**
**04.10.2011**	**A/duck/Moscow/4521/2011**	**d/4521/11**	**H3N8**
**04.10.2011**	**A/duck/Moscow/4522/2011**	**d/4522/11**	**H3N8**
**10.10.2012**	**A/duck/Moscow/4772/2012**	**d/4772/12**	**H4N6**
